# Role of Phenolic Acids from the Rhizosphere Soils of *Panax notoginseng* as a Double-Edge Sword in the Occurrence of Root-Rot Disease

**DOI:** 10.3390/molecules23040819

**Published:** 2018-04-02

**Authors:** Ya-Meng Zhao, Yong-Xian Cheng, Yu-Nan Ma, Chuan-Jiao Chen, Fu-Rong Xu, Xian Dong

**Affiliations:** 1 College of Pharmaceutical Sciences, Yunnan University of Traditional Chinese Medicine, Kunming 650500, China; yamengzhao108@163.com (Y.-M.Z.); yxcheng@szu.edu.cn (Y.-X.C.); mayunan994727@163.com (Y.-N.M.); chenchuanjiao663@163.com (C.-J.C.); 2 Guangdong Key Laboratory for Genome Stability & Disease Prevention, School of Pharmaceutical Sciences, Shenzhen University Health Science Center, Shenzhen 518060, China

**Keywords:** *Panax notoginseng*, rhizosphere soils, phenolic acids, fusaric acid, *Fusarium oxysporum*

## Abstract

Chemical agents in the rhizosphere soils of plants might have an influence on root-rot disease, which therefore might reveal the mechanism of root rot in *Panax notoginseng* (*P. notoginseng*). With this hypothesis the alterations of phenolic acids (PAs) in the rhizosphere soils of *P. notoginseng* after pathogen infection were determined. The effects of PAs on the growth of *Fusarium oxysporum* (*F. oxysporum*), a fungal pathogenic factor for *P. notoginseng*, as well as production of fusaric acid, a wilting agent for the plants, were also examined. The results indicate the presence of five PAs (ferulic acid, syringic acid, *p*-hydroxybenzoic acid, *p*-coumaric acid, and vanillic acid) in the rhizosphere soils of *P. notoginseng*, whose contents in the rhizosphere soils of healthy plants are higher than those of the diseased ones. Further we found that individual PA could inhibit the mycelium growth and spore production of *F. oxysporum*, but stimulate fusaric acid production as well, disclosing the double-edge sword role of PAs in the occurrence of root rot of *P. notoginseng* and paving the way for the intervention of *P. notoginseng* root rot via balancing PAs.

## 1. Introduction

The root of *Panax notoginseng* (Araliaceae), known as San-Qi in Chinese, is a common and prominent traditional Chinese medicine due to its hemostatic and restorative properties. *P. notoginseng* is mainly spread over southwestern China, with Wenshan County of Yunnan Province being known as the main producing area [1]. The remarkable curative effects of San-Qi on cardiovascular diseases has resulted in a great demand far exceeding wild supply. As a consequence, cultivation of San-Qi has been documented for over 400 years [1]. Cultivation of San-Qi meets the demand, however, one problem arising from scaled-up cultivation is root rot, which is now a bottleneck limiting further development of the San-Qi industry. Root-rot disease could be caused by bacteria and nematodes, but it mainly results from fungal pathogens such as *Fusarium* and *Cylindrocarpon* species [2]. *Fusarium solani* and *Fusarium oxysporum* are highly aggressive fungi for San-Qi root rot in the producing region of China. The typical early stage symptoms of this disease are reddish-brown to orange-brown discolored areas on the root surface. As the disease develops, other symptoms include dry rot in both exterior and interior root tissues, and loss of fibrous roots. The infection of the root tissues might be associated with changes in the ginsenoside contents [3]. *Fusarium* species could infect the plant through the root system, then colonize root system and lower stem, and finally result in the disease [4]. Toxins generated by fungi in the process of invading plants, could interfere with the metabolism of the host plant. Among them, fusaric acid (FA) produced by *F. oxysporum* is a non-specific toxin [5,6], which could interfere with the metabolic functions of plants within the physiological concentration range. The ground part disease of *P. notoginseng* can be prevented and controlled in advance through observing the changes to the leaves, however, the underground part of the plant disease can’t be easily found and is hard to control. To some extent, root rot has become the most serious disease [7].

The concept of allelopathy was first put forward by Molisch in 1937. Allelochemicals are mainly secondary metabolites in plants arising by secondary metabolism, exemplified by the acetic acid or shikimic acid pathway, which have an influence on plant growth. So far extensive investigations have been conducted on allelochemicals [8,9]. The sensitive substances were mainly released into the environment through leaching of rain and fog, natural volatilization, root secretion, and plant decomposition, which could affect the growth of plants. For *P. notoginseng*, the main limiting factor of production is the continuous cropping obstacle caused by the change of soil microbial community (pathogenic microorganisms increased and beneficial microbes decreased), the self-toxic effects caused by chemical substances, and changes of soil nutrients and other physical and chemical properties. Considering that phenolic acids (PAs) are important allelochemicals that cause continuous cropping obstacles, this study was designed to observe the types and content of PAs in the rhizosphere soils under continuous cropping field conditions, the effects of PAs on *F. oxysporum* growth in in vitro experiments, and finally reveal the possible mechanisms of Pas’ effect in root-rot disease of *P. notoginseng*.

## 2. Results

### 2.1. Determination of PAs in Soils

**Figure 1:**
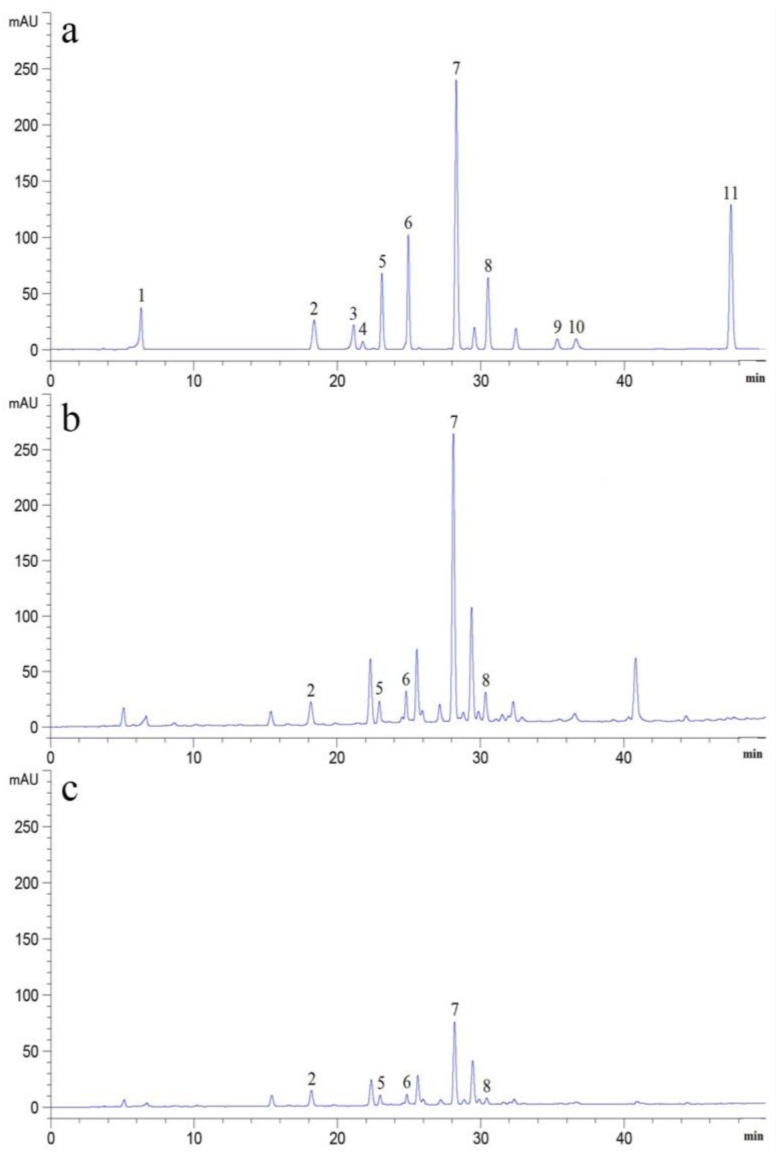
Chromatogram of PAs from rhizosphere soil of *P. notoginseng.* Notes: (**a**) PAs standards; (**b**) PAs in rhizosphere soil of healthy root; (**c**) PAs in rhizosphere soil of disease root. 1: Gallic acid; 2: *p*-Hydroxybenzoic acid; 3: Chlorogenic acid; 4: *o*-Phthalic acid; 5: Vanillic acid; 6: Syringic acid; 7: *p*-Coumaric acid; 8: Ferulic acid; 9: Benzoic acid; 10: Salicylic acid; 11: *t*-Cinnamic acid.

**Figure 2:**
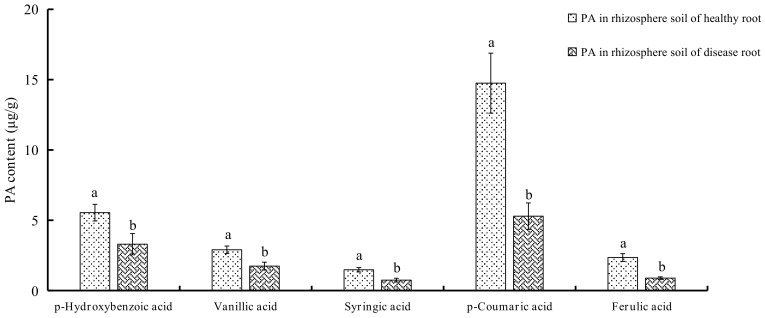
The content of PA in rhizosphere soil of both healthy and disease *Panax notoginseng* (μg/g). Each data point represents the mean ± SD of eight replicates. Different letters represent significant differences (*P* < 0.05) between rhizosphere soil of healthy root and disease root.

The types and content of PAs in soil samples are shown in Figure 1 and Figure 2. In this study, five Pas, including *p*-hydroxybenzoic acid, vanillic acid, syringic acid, *p*-coumaric acid, and ferulic acid were detected in the rhizosphere soils of *P. notoginseng*. To our surprise, levels of all these PAs were much higher in rhizosphere soils of healthy plants than those of diseased ones (*P* < 0.05) with *p*-coumaric acid being the highest (14.7502 μg/g), exceeding by 2.79-fold that found in rhizosphere soils of diseased roots. *p*-Hydroxybenzoic acid was the second highest, with a concentration of 5.5405 μg/g in the rhizosphere soils of healthy roots but 3.2969 μg/g in the diseased ones. In contrast, the abundance of ferulic acid, vanillic acid, and syringic acid in rhizosphere soils of healthy plants is not as high as that of *p*-coumaric acid or *p*-hydroxybenzoic acid but higher than that of diseased ones. As far as the total PAs are concerned, the content in the rhizosphere soils of healthy plants is 63.3667 μg/g versus 38.0500 μg/g in the rhizosphere soils of diseased roots.

### 2.2. Growth Inhibition of F. oxysporum

**Figure 3:**
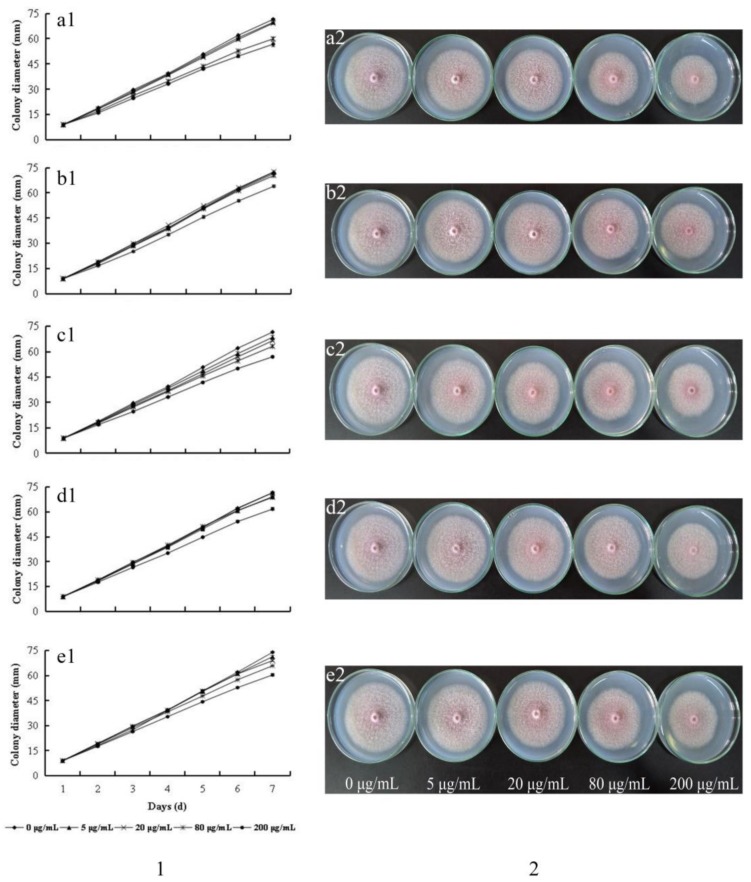
Effects of different concentrations of PAs on the growth of *F. oxysporum*. Notes: (**1**) The growth curve of *F. oxysporum*; (**2**) Effects on the growth of *F. oxysporum* after 7 days culture. (**a**) Ferulic acid; (**b**) Syringic acid; (**c**) *p*-Hydroxybenzoic acid; (**d**) *p*-Coumaric acid; (**e**) Vanillic acid.

As shown in Figure 3, individual PA could inhibit the growth of *F. oxysporum* and the colony diameter decreased with the increase of PA concentration in the medium. The strongest inhibitory effect was observed for ferulic acid. In contrast, the other PAs such as *p*-hydroxybenzoic acid, vanillic acid, *p*-coumaric acid and syringic acid showed weak inhibitory activity. At 5 μg/mL, the inhibitory effect of ferulic acid was observed in 2-day incubation. However, no further significant difference was detected with the growth of pathogen. When the concentration of phenolic acids reaches up to 80 μg/mL, their inhibitory effect presents significant difference (*P* < 0.05) compared with the control group.

### 2.3. Determination of Spore Yields of F. oxysporum

**Figure 4:**
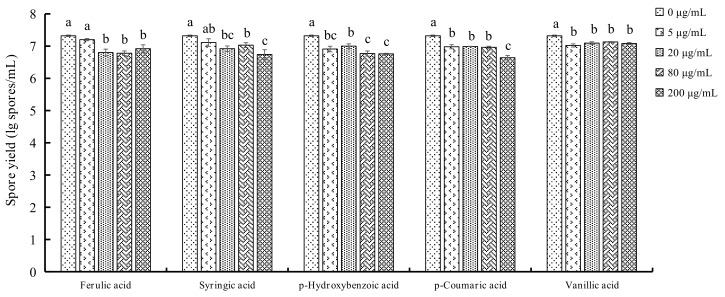
Effects of different PAs concentrations on the spore yield of *F. oxysporum* (lg spores/mL). Each data point represents the mean ± SD of five replicates. Different letters represent significant differences (*P* < 0.05) among different PAs concentrations.

The effects of different concentrations of PAs on the spore yields of *F. oxysporum* are shown in Figure 4. It be seen that PAs significantly reduce the spore yields of *F. oxysporum* at 5 μg/mL. The most reduction is 58.71% compared with control observed in the *p*-hydroxybenzoic acid treatment. The second highest reduction is 53.19% in the *p*-coumaric acid treatment. With the increasing concentration, the spore yields decline for ferulic acid, syringic acid, *p*-hydroxybenzoic acid, and *p*-coumaric acid treatment and the strongest inhibition is observed when the concentration is up to 200 μg/mL.

### 2.4. FA Production in Both F. oxysporum and P. notoginseng

**Figure 5:**
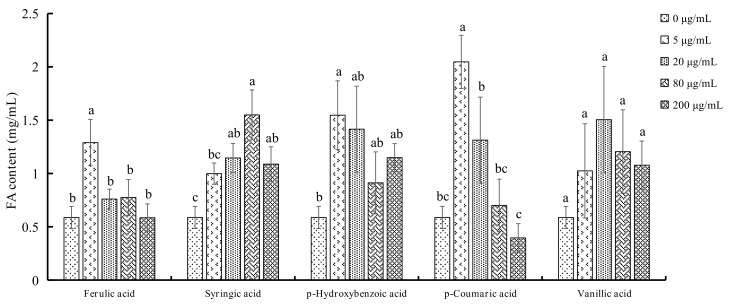
Influence of different concentrations of PAs in the production of FA in *F. oxysporum*. Each data point represents the mean ± SD of five replicates. Different letters represent significant differences (*P* < 0.05) among different PAs concentrations.

The effect of different concentrations of PAs on FA production is shown in Figure 5. It was found that 5 μg/mL of ferulic acid, *p*-hydroxybenzoic acid, and *p*-coumaric acid induce 2.20, 2.66, and 3.52-fold higher FA production compared with control, respectively. For syringic acid treatments, FA production increases all FAs compared with the control and reaches the highest level at 80 μg/mL. In addition, it was found that vanillic acid has no influence on FA generation even at the 200 μg/mL. 

### 2.5. Effects of FA on the Growth of P. notoginseng

As shown in Table 1, the wilting rates, the disease index and FA content in the plants increase with the increased concentration of FA. The wilting rates are 13.33%, 16.67%, and 20.00%, respectively, corresponding to FA concentrations of 50, 100, and 200 ppm. The disease index of the plants is as high as 16.68 at 200 ppm of FA. FA could be detected in all the three treatments and the highest level is 36.56 μg/g FW when the FA concentration is 200 ppm, which represents a 6.83-fold increase compared with that observed at 50 ppm. 

## 3. Materials and Methods

### 3.1. Soil Materials

Soil samples were collected from the village of Xiao Secong, in Shizong County, Qujing City, Yunnan province, China. Four-year-old *P. notoginseng* were divided into healthy and diseased plants through observing both the ground parts and roots, which were used to collect the rhizosphere soils. Briefly, the plants of each plot were uprooted and gently shaken with hands to remove all loosely adhering soils. The rest of soils adhering could not be shaken off the root surface, was removed with sterilized hairy brush considered as rhizosphere soils [10]. All the samples were performed in eight replicates. The soil sample were sieved into 20 mesh screen to remove the fibrous roots and gravels, and then stored at −20 °C until use. 

### 3.2. Indicator Fungi

The tested pathogenic fungi, isolated from the roots of infected *P. notoginseng*, were identified as *F. oxysporum* by Sangon Biotech (Shanghai) Co., Ltd. (Shanghai, China). Vigorous strains were used in the further experiments after 3–4 rounds of activation on PDA medium.

### 3.3. Determination of PAs in Soil Samples

Ten g of fresh soil were weighed into a 50 mL centrifuge tube and 25 mL NaOH (2M) was added. The tube was then placed on an oscillator at 120 rpm, 30 °C for 24 h and centrifuged at 10,000 rpm for 10 min. The supernatant was adjusted to pH 2.5 using 5 N HCl, and extracted three times with ethyl acetate. The organic phase was combined and evaporated to dryness at 35 °C on an evaporator. The residue was dissolved in 3 mL 80% methanol and maintained in the dark at 4 °C. The sample should be filtered through 0.45 μm organic filter membrane before HPLC injection [11,12]. 

The standard PAs used for analysis are gallic acid, *p*-hydroxybenzoic acid, chlorogenic acid, *o*-phthalic acid, vanillic acid, syringic acid, *p*-coumaric acid, ferulic acid, benzoic acid, sallcylic acid, *t*-cinnamic acid, which were bought from Kunming Youning Technology Co., Ltd. (Kunming, China). The compounds were identified using an HPLC system (Agilent 1260II, Santa Clara, CA, USA). The analytical conditions [13] were as follows: chromatographic column: XDB-C18 (250 × 4.6 mm, 5 μm, i.d., Agilent), temperature: 35 °C, flow rate: 0.8 mL/min, detection wavelength: 280 nm, injection volume: 10 μL. Methanol (A) and 2% acetic acid (B) were used as mobile phases with a gradient elution (0–15 min: 5–15% A; 15–20 min: 15–30% A; 20–40 min: 30–45% A; 40–50 min: 45–60% A; 50–51 min: 60–90% A; 51–65 min: 90% A; post run 20 min). All the calibration curves of 11 PAs exhibit good linearity (*r*^2^ > 0.99) in the concentration ranges investigated in this study. 

The total PAs of rhizosphere soils was determined by using the Folin-Ciocalteu method. One mL of the above supernatant was added to the 25 mL volumetric flask containing 9 mL distilled water. One mL double-distilled H_2_O was prepared as a reagent blank. One mL of Folin-Ciocalteu’s phenol reagent was added to the mixture and thoroughly shaken for 30 s. After 5 min, 10 mL of 7% Na_2_CO_3_ solution was added to the mixture. Then the solution was diluted to 25 mL with double-distilled H_2_O and mixed. After incubating for 90 min at room temperature, the absorbance was determined at 765 nm using a UV spectrophotometer. Total PAs of rhizosphere soils was calculated according to a gallic acid standard curve. The PAs content was expressed as mg·g^−1^ FW. 

### 3.4. Determination of F. oxysporum Growth

The effects of phenolic acid on *F. oxysporum* growth were determined by growing the fungus on plates containing different amounts of PAs. PA samples were dissolved in 50% methanol and added to the medium. Various concentrations of PAs detected above were added to 1/2 PDA culture medium before it had solidified in standard 90-mm Petri dishes containing a total volume of 15 mL. The final concentrations of PA in the agar plates were 0, 5, 20, 80 and 200 μg/mL, respectively. 5-mm-diameter discs of fungus-containing agar was inoculated to the center of medium and cultured for 7 days at 28 °C. The diameter of the colony was measured per 24 h with a ruler until the 7th day [14].

### 3.5. Determination of the Spore Yields of F. oxysporum

After measuring the growth of *F. oxysporum* on 1/2 PDA medium above for 7 days, 30 mL sterile water was added to the Petri dishes to obtain all the fungal culture with an aseptic slide. The resulting fungal cultures obtained were filtered through four layers of cheesecloth to remove the mycelia and then centrifuged at 8000× *g* for 20 min to pellet the conidia. The conidia were resuspended in sterile water and quantified using a hemocytometer. 

### 3.6. Determination of FA in Both F. oxysporum and P. notoginseng

To determine the effects of PAs on FA production, *F. oxysporum* was inoculated into Czapek Dox medium (50 mL in 100-mL flasks) amended with PAs. Five different concentrations of PAs culture medium were prepared. The treatments were 0, 5, 20, 80 and 200 μg/mL, respectively. The 200 μL spore suspension (5.35 × 10^6^ spores/mL) was added to the Czapek medium. The pathogen was incubated at 28 °C on a rotary shaker (180 rpm) for about 10 days [15]. The culture was filtered with a 0.45 μm membrane to exclude mycelia and microconidia. Subsequently, the filtrate was adjusted to pH 2.5 with 2 M HCl and extracted three times with an equal volume of methylene chloride. The organic phase was pooled and lyophilized under a vacuum. The residue was dissolved in 3 mL of methanol to obtain the crude toxin solution. 

FA content in plant was measured according to Smith’s method [16]. The tissues were weighed and homogenized in a juice extractor with MeOH/1% KH_2_PO_4_ (1:1, *v*/*v*, pH 2.5). The suspension was then centrifuged at 10,000× *g* for 15 min. The clarified supernatants were pooled and the pH of the supernatant was adjusted to 2.5 with 2 M HCl. The acidified supernatant was sequentially extracted with 50 mL of methylene chloride. The methylene chloride extracts were pooled and evaporated to dryness at 45 °C on a rotary evaporator. The residue was redissolved in 3 mL of MeOH and stored at −20 °C until HPLC use as described above. The content of FA was expressed as μg·g^−1^ FW. 

The content of FA was determined by HPLC using a XDB-C18 column (250 × 4.6 mm, 5 μm, i.d., Agilent), temperature: 35 °C, flow rate: 1 mL/min, detection wavelength: 273 nm, injection volume: 1 μL, methanol (A) and 0.43% phosphoric acid (B) were used as mobile phases under isocratic elution (68:32), operation time was 15 min [17].

### 3.7. The Effect of FA on the Growth of P. notoginseng

Different concentrations (0, 50, 100, and 200 ppm) FA (Sigma, St. Louis, MO, USA) were prepared. 120 plants of the same appearance were selected to immerse into FA solution. The plants were graded for severity of FA treatment as 0 (not showing wilting symptom), 1 (the stem soft), or 2 (the stem fall, but the leaf not wilted) and 3 (the plant wilting completely). The wilting rate and the disease index were calculated:

The wilting rate (%) = the number of wilting plants/total number of plants ×100

Disease index = ∑ (number of plants rated × rating)/(total number of plants× highest rating) × 100 [18]. 

### 3.8. Statistical Analysis

Differences among the treatments were calculated and statistically analyzed using the analysis of variance (ANOVA) feature of the SPSS software (Analytical Software, Tallahassee, FL, USA). Differences between treatments were determined, and *P* < 0.05 was taken to indicate statistical significance.

## 4. Discussion

*Panax notoginseng* possesses excellent therapeutic effects on cardiovascular and cerebrovascular diseases. The planting area is expanding with the increasing demand. However, the continuous cropping obstacles of *P. notoginseng* could lead to less land being available to cultivate *P. notoginseng*. Root rot has been responsible for major losses in *P. notoginseng* production. *Fusarium* species can survive in plant debris or in the soils through resistant structures such as chlamydospores for a long time, or in the form of spores or mycelium-infected or dead tissue. The plant rhizosphere is an important ecological environment in the soil for plant-microbe interactions. These interactions with plants could be beneficial, neutral or detrimental, resulting in plant diseases [19]. Phenolics are widely distributed in plants and there are often large increases in phenolic synthesis in plants after infection with plant pathogens [20]. Phenolics-based defense responses are characterized by an early accumulation of phenolic compounds at the infection site [21]. The aim of the current work was to identify changes in the levels of phenolic acids in the rhizosphere after pathogen infection and its effect on *F. oxysporum*. 

It was reported that six PAs were detected in rhizosphere soil of *P. notoginseng*. They were *p*-hydroxybenzoic acid, vanillic acid, syringic acid, *p*-coumaric acid, ferulic acid and benzoic acid, of which the highest content was *p*-coumaric acid, and the lowest content was syringic acid. In our study, only five PAs were detected, and the identity of the PAs found at the highest and lowest level were consistent with the results of the previous study. There are few studies to compare the change of PAs between rhizosphere soils of healthy and infected plants. In our study, it was found that after pathogen infection, individual PAs and total content of PAs decreased (Figure 1 and Figure 2). This could be the results of an allelopathic effect of plant metabolites affected by the soil microbial community [22,23], and PAs catabolised by pathogena could lead to the decreased amounts. The content of the detected PAs decreased in the rhizosphere soils of infected *P. notoginseng* (Figure 2), which allows us to speculate that Pas, as substances of the defense response of *P. notoginseng*, could reduce both the mycelium growth and spore production. As the infection persists, PAs could be transformed into other resistant substances by the activity of phenylalanine ammonia-lyase (PAL) and peroxidase (POD) [24,25] and correspondingly the secretion into rhizosphere soils was less than during the early stage. A previous study indicated that adding PA to soils could reduce *Fusarium* wilt of cucumber, and the effect was strengthened with the increased concentration (50–1000 mg/kg soils), and the relative potency is *p*-hydroxybenzoic acid > coumaric acid > ferulic acid [26]. The cause why PAs reduce disease includes the fact that PAs change the composition of microfiora in soils and reduce the proportion of pathogen. High concentration of PAs could inhibit the growth of pathogen mycelium, and improve the ability of soil actinomycetes to antagonize pathogen. Growth of *F. oxysporum* in an in vitro experiment by exogenous addition PAs were determined in our study. PA could inhibit the growth of *F. oxysporum* (Figure 3) and the inhibition rate is proportional to the concentration of PAs. The inhibition rate reaches the highest value at a concentration of 200 μg/mL. The inhibitory potency order is ferulic acid > *p*-hydroxybenzoic acid > vanillic acid > *p*-coumaric acid > syringic acid. Compared with control, spore yields decrease with added exogenous PAs (Figure 4). At 5 μg/mL, the spore yields are also reduced by 23.33%, 34.09%, 58.71%, 53.19%, 49.11%, respectively, for ferulic acid, syringic acid, *p*-hydroxybenzoic acid, *p*-coumaric acid, and vanillic acid. In contrast, at 200 μg/mL, significant declines are observed, decreasing by 70.24%, 72.16%, and 78.54% for syringic acid, *p*-hydroxybenzoic acid, and *p*-coumaric acid, respectively. The process of infection by *F. oxysporum* could be divided into germination and adhesion of infection hyphae to the root, invasive growth on the root cortex, hyphal growth within the xylem vessels and production of microconidia, and finally invasion of the moribund plant tissue and production of chlamydospores [27]. As long as the plant is alive, the defense response still exists. Root exudates are supposed to play a key role in determining the positive or negative outcome of an interaction in the rhizosphere. In the present study, PAs secreted by roots were released into the rhizosphere. 

Wilt disease is most likely caused by a combination of pathogen activities, such as the accumulation of fungal mycelium and/or toxin production and host defense responses, including production of gels, gums and tyloses and vessel crushing by proliferation of adjacent parenchyma cells. Although PAs detected in our study could reduce the mycelium growth and spore production, they stimulated the toxin production to a large extent (Figure 5). For ferulic acid, *p*-hydroxybenzoic acid, and *p*-coumaric acid, FA increases by 2.20, 2.63, 3.48 folds compared with control when the concentration was 5 μg/mL. In contrast, 80 μg/mL syringic acid and 20 μg/mL vanillic acid lead to FA production increases by 2.64- and 2.56-fold, respectively. FA, a toxin produced by *Fusarium* species, is one of the first fungal metabolites implicated in the pathogenesis of wilt disease. The wilt rate and disease index of *P. notoginseng* plants increase with elevated FA concentration in plants (Table 1). A study [14] indicated that cinnamic acid could inhibit the growth of pathogens including colony diameter, conidial germination, sporulation, biomass production and enzyme activity in the concentration ranging from 0 mg/L to 1600 mg/L. In addition, at the concentration up to 1600 mg/L, the complete inhibition was observed. However, it stimulated the production of toxin. The results are consistent with our findings. Ferulic acid also inhibited the the growth of *P. notoginseng* pathogens (*Cylindrocarpon destructans* (zinss.) Scholton, *Fusarium solani and Alternaria panax* Whetzel) in vitro. In addition, the inhibition effect became stronger with the concentration increased, ranging from 0 mg/kg to 2000 mg/kg [28]. There was a study showing that *p*-coumaric acid not only suppresses *Fusarium* sporulation and spore germination directly within limits in a dose dependent manner, but also enhances watermelon resistance to *Fusarium* by enhanceing chitinase activity, β-1,3-glucanase activity, and *CIPR3* expression [29]. There is a study indicated that syringic acid was found to exhibit significant antifungal activity (*P* < 0.001) in *Fusarium moniliforme*, *Fusarium pallidoroseum*, *Curvularia lunata* and *Helminthosporium* sp by inhibiting spore germination compared to control, whose percentage of spore germination was 71%, 75%, 70% and 71%, respectively. As the concentration rised, the inhibition activity was strengthened, and the spore germination rates were decreased by 36.62–43.66%, 44.00–46.67%, 28.57–37.14%, and 50.70–61.97% within the concentration range 500 ppm to 1000 ppm for *F. moniliforme*, *F. pallidoroseum*, *C. lunata* and *Helminthosporium* sp. Respectively, compared with control, [30]. Vanillic acid at the concentration of 0.1–1 mmol/L stimulated the growth of *Rhizoctonia solani*, but did not change with concentration. It was significantly promoted (*P* < 0.05) compared with control when the concentration was 0.5 mmol/L. For *Fusarium solani*, vanallic acid was characterized by low concentration promotion and high concentration inhibition. When the concentration reached 1.0 mmol/L, the growth of *F. solani* was inhibited.

The effects of allelochemicals are complex, mainly including synergistic and antagonistic effects. In the actual circumstances, the concentration of some allelochemicals is low, which cannot influence plants, but allelopathy actually occurs. Also, another reason might be that single allelochemical could work together with other existing chemical components. Under laboratory conditions, research was conducted using a single phenolic acid, which is inconsistnt wiath the actual field conditions of *P. notoginseng*. From our present results, we speculate that high concentrations of these kinds of PAs could reduce pathogen growth, whereas low concentrations could increase the pathogenesis of pathogens by increasing toxin production, so plants producing less PAs would be more susceptible to infection. The occurrence of root rot is a consequence of various factors, and might also be a combination of various PAs, so the specific mechanism of action needs to be further studied.

## 5. Conclusions

This study reveals that PAs detected in rhizosphere soils of *P. notoginseng* with 3-year continuous cropping are ferulic acid, syringic acid, *p*-hydroxybenzoic acid, *p*-coumaric acid, and vanillic acid. After pathogen infection, the content of PAs decreases compared with non-infected plants. Individual PA could decrease the mycelium growth, spore production of *F. oxysporum* but stimulate FA production, especially at low concentrations. Since wilt disease of *P. notoginseng* is associated both with *F. oxysporum* and FA, our present study illustrates an example of a double-edge sword role of PAs in the farming of *P. notoginseng*.

## Figures and Tables

**Table 1:** Effects of the FAs on the growth of *P. notoginseng* plants.

Concentration (ppm)	Wilting Rate (%)	Disease Index	FA Concent in Plants (μg/g)
0	0.00 ^b^	0.00 ^c^	0 ^c^
50	13.33 ± 3.33 ^a^	6.66 ± 1.93 ^b^	5.3571 ± 0.2572 ^bc^
100	16.67 ± 3.33 ^a^	11.11 ± 2.22 ^b^	15.0364 ± 3.9238 ^b^
200	20.00 ^a^	18.89 ± 1.11 ^a^	36.5687 ± 5.9505 ^a^

Note: Different letters in the same column represent significant differences (*P* < 0.05) among different FA concentrations.
